# A Machine Learning Approach to Predicting Autism Risk Genes: Validation of Known Genes and Discovery of New Candidates

**DOI:** 10.3389/fgene.2020.500064

**Published:** 2020-09-10

**Authors:** Ying Lin, Shiva Afshar, Anjali M. Rajadhyaksha, James B. Potash, Shizhong Han

**Affiliations:** ^1^Department of Industrial Engineering, University of Houston, Houston, TX, United States; ^2^Division of Pediatric Neurology, Department of Pediatrics, Weill Cornell Medicine, New York, NY, United States; ^3^Feil Family Brain & Mind Research Institute, Weill Cornell Medicine, New York, NY, United States; ^4^Weill Cornell Autism Research Program, Weill Cornell Medicine, New York, NY, United States; ^5^Department of Psychiatry and Behavioral Sciences, Johns Hopkins School of Medicine, Baltimore, MD, United States; ^6^Lieber Institute for Brain Development, Baltimore, MD, United States

**Keywords:** autism, *de novo* mutation, gene expression, constraint, machine learning

## Abstract

Autism spectrum disorder (ASD) is a complex neurodevelopmental condition with a strong genetic basis. The role of *de novo* mutations in ASD has been well established, but the set of genes implicated to date is still far from complete. The current study employs a machine learning-based approach to predict ASD risk genes using features from spatiotemporal gene expression patterns in human brain, gene-level constraint metrics, and other gene variation features. The genes identified through our prediction model were enriched for independent sets of ASD risk genes, and tended to be down-expressed in ASD brains, especially in frontal and parietal cortex. The highest-ranked genes not only included those with strong prior evidence for involvement in ASD (for example, *NBEA*, *HERC1*, and *TCF20*), but also indicated potentially novel candidates, such as, *MYCBP2* and *CAND1*, which are involved in protein ubiquitination. We also showed that our method outperformed state-of-the-art scoring systems for ranking curated ASD candidate genes. Gene ontology enrichment analysis of our predicted risk genes revealed biological processes clearly relevant to ASD, including neuronal signaling, neurogenesis, and chromatin remodeling, but also highlighted other potential mechanisms that might underlie ASD, such as regulation of RNA alternative splicing and ubiquitination pathway related to protein degradation. Our study demonstrates that human brain spatiotemporal gene expression patterns and gene-level constraint metrics can help predict ASD risk genes. Our gene ranking system provides a useful resource for prioritizing ASD candidate genes.

## Introduction

Autism spectrum disorder (ASD) is a neurodevelopmental condition characterized by impaired social interaction and communication, as well as repetitive behavior. While its etiology is complex, ASD has a strong genetic basis (Hallmayer et al., 2011; Jeste and Geschwind, 2014; Colvert et al., 2015). The role of *de novo* mutations in ASD has been firmly established through candidate gene (Wang et al., 2016; Stessman et al., 2017), whole exome (Iossifov et al., 2012, 2014; Sanders et al., 2012; Ronemus et al., 2014), and whole genome sequencing studies (Ryan et al., 2017; Turner et al., 2017). Although the list of risk genes implicated by *de novo* mutations is growing, it is still very likely far from complete, with an estimated full set of ASD genes ranging from several hundred to more than 1,000 (Iossifov et al., 2014). In the search for additional *de novo* mutations, sequencing studies continue to be an important approach, but the current sequencing cost is still very high, especially for large samples. As an alternative strategy, advanced analytical approaches, which leverage previously implicated genes and prior knowledge, have the potential to enhance risk gene discovery in an efficient and cost-effective manner (Asif et al., 2018; Gök, 2018; Brueggeman et al., 2020).

One approach is based on the concept of guilt-by-association, i.e., assuming that genes that confer risk for ASD are likely to be functionally related, and that they thus converge on molecular networks and biological pathways implicated in disease (Gandhi et al., 2006; Xu and Li, 2006). For example, one study showed that ASD genes with *de novo* mutations converged on pathways related to chromatin remodeling and synaptic function (Krumm et al., 2014). To leverage these functional relationships, several studies have explored integrating known risk genes using a protein-protein interaction (PPI) network to identify novel genes involved in ASD (Gilman et al., 2011; Li et al., 2014; Hormozdiari et al., 2015; Liu et al., 2015). However, a PPI network is built upon general PPIs without reference to tissue or cell type specificity, and this approach may not fully capture the brain-centric functional relationships among ASD genes. Accordingly, a brain-specific network-based approach, which considered relationships within the context of the brain, was proposed to predict ASD genes (Krishnan et al., 2016; Duda et al., 2018). Studies employing this paradigm, however, did not consider the dynamic patterns of gene relationships during brain development, thereby limiting their potential for discovery, given the possibility that genes might only be functionally related within a specific developmental stage. Evidence for this comes from Willsey et al. (2013) who showed, using spatiotemporal gene expression data from human brain, that co-expression patterns of ASD risk genes varied by spatiotemporal windows, with the strongest co-expression patterns observed in the prefrontal and primary motor–somatosensory cortical regions during midfetal development, suggesting an important convergence of risk gene activity in particular places at a particular time.

In addition to having functional relationships, ASD genes affected by *de novo* mutations tend to be intolerant of variations (Samocha et al., 2014; Iossifov et al., 2015). With the availability of sequencing data from large samples, recent work has developed measures to quantify the sensitivity of genes to disruptive functional variations (Petrovski et al., 2013; Lek et al., 2016). Utilizing exome data on more than 60,000 individuals from the Exome Aggregation Consortium (ExAC), a gene-level constraint metric–the probability of being loss-of-function (LoF) intolerant (pLI)–was created, which separates genes into LoF intolerant or LoF tolerant (Lek et al., 2016). Kosmicki et al. (2017) further demonstrated that the excess of *de novo* mutations in ASD individuals was primarily driven by LoF-intolerant genes, but not LoF-tolerant genes.

We reasoned that ASD risk genes show expression patterns that are clustered in specific brain regions and developmental stages critical to disease development, and that high resolution spatiotemporal gene expression patterns in human brain can help distinguish genes that cause disease from those that do not. In addition, because ASD genes affected by *de novo* mutations are sensitive to mutational changes, we reasoned that gene-level constraint metrics can further differentiate ASD genes from normal ones. The objective of this study was to employ a machine learning-based approach to predict ASD risk genes using human brain spatiotemporal gene expression signatures, gene-level constraint metrics, and other gene variation features. We compared the performance of our method with five other state-of-the-art scoring systems for ranking ASD candidate genes, and evaluated the risk genes from our prediction model using independent sets of risk genes and differential gene expression (DGE) evidence. Gene Ontology (GO) enrichment analysis was also performed to understand the biology underlying ASD risk genes.

## Materials and Methods

### Gene Set

To train the gene prediction model, we used labeled genes curated by Duda et al. (2018) as described in detail elsewhere. Briefly, the labeled genes contained 143 true positive genes and 1,145 true negative ones. The true positives came from the high confidence genes in the Simons Foundation Autism Research Initiative (SFARI) resource^1^ (Category 1, Category 2, and syndromic genes) and the 65 reported genes in Sanders et al. (2015). The true negative genes were selected from the non-ASD gene list created by Krishnan et al. (2016), which were genes associated with non-mental health diseases, as annotated in OMIM. Among these genes we focused on those that had both gene expression data from the BrainSpan atlas and gene-level constraint metrics available, so that our final training gene set included 121 true positive genes and 963 true negatives.

### Prediction Feature Sets

The feature sets in our prediction task included spatiotemporal gene expression patterns in human brain, network features, gene-level constraint metrics, and other gene variation features. Supplementary Table S1 provides a summary of all features. We provide details below for each feature set.

### Spatiotemporal Gene Expression

We downloaded RNA-Seq data (version 10), summarized to Gencode v20 gene-level reads per kilobase per million mapped reads (RPKM) values, from the BrainSpan website^2^. Detailed information on tissue processing, experimental and bioinformatics procedures related to the RNA-Seq data is available at the BrainSpan website. The BrainSpan dataset includes 524 gene-level expression features for each gene across 13 developmental stages in 31 brain regions from 524 brain samples spanning a variety of developmental stages and brain regions. Gene expression values were log-transformed (log_2_ [RPKM + 1]) and were used to predict autism genes.

To capture the functional relationships among genes, we built a weighted network for genes with both gene co-expression and PPI evidence from InWeb (Rossin et al., 2011). Specifically, the co-expression level between a gene pair was assessed by the Fischer z-transformed Pearson correlation between their spatiotemporal gene expression values. The genes with PPIs were connected and their edges were weighted by their co-expression levels. We extracted a set of network features that characterized the network topologies using *igraph* package in R. Specifically, we measured the node centralities using node degrees, clones centralities, betweenness centralities, Bonacich power centralities, eigenvector centralities, and alpha centralities (Bonacich, 1987). We captured the modules in functional relationship networks using the principle component decomposition and K-core decomposition (Batagelj and Zaversnik, 2003). The loading of the 1st principle component, hub score and coreness were obtained for each node. The importance of each node was further measured using the PageRank algorithm (Brin and Page, 1998), which counts the number and weight of links to each node. In total, 10 network features were extracted from the weighted gene network and were used for autism risk gene prediction. For genes appeared in BrainSpan but not in PPI network, we imputed their network features using the k-Nearest Neighbor algorithm.

### Gene-Level Constraint Metrics and Other Gene Variation Features

We used gene-level constraint metrics developed from the exome data of more than 60,000 individuals from the ExAC to quantify the sensitivity of genes to variations (25). We considered six gene-level constraint metrics, including Z scores for synonymous (syn_z), missense (mis_z), and LoF variants (lof_z), the pLI, the probability of being intolerant of homozygous but not heterozygous LoF variants (pRec), and the probability of being tolerant of both heterozygous and homozygous LoF variants (pNull). A higher Z score or pLI indicates that the gene is more intolerant of variation (more constrained). We also included 10 general gene features, including the number of coding base pairs (bp), probabilities of mutations across the transcript for synonymous (mu_syn), missense (mu_mis), and LoF variants (mu_lof), number of rare variants (n_syn, n_mis, n_lof), and depth adjusted number of expected rare variants (exp_syn, exp_mis, exp_lof). Gene-level constraint metrics and general gene features were downloaded from the ExAC website^3^. Wilcoxon rank sum test was used to compare the group differences in above features between known ASD risk and non-risk genes.

### Autism Risk Gene Prediction

We used machine learning methods to predict autism risk genes from their spatiotemporal expression signatures, network topology features, gene-level constraint metrics, and other general gene features. We applied four machine learning methods ranging from ones that are regression based [logistic regression and support vector machines (SVM) with Gaussian kernel] to others that are tree based (random forest and gradient boosted trees). The gradient boosted trees model ensembles a set of trees for prediction bias reduction and was trained in the XGBoost package (Chen et al., 2015). The optimal tuning parameters in each model were selected by a nested grid-search, and model performances were evaluated by five-fold cross validation (CV) on training data. The prediction accuracy was measured by the area under the receiver-operator curve (AUC-ROC) on the hold-out set for each fold of the CV. As the training data is unbalanced with small number of autism risk genes, we further considered the area under precision-recall curve (AUC-PRC) to measure the prediction accuracy.

Based on the average prediction accuracy over five folds, the gradient boosted trees model (BTree) was selected as the optimal algorithm. The final prediction model was built by applying the gradient boosted trees algorithm (with optimally tuned parameters) on all training genes and stored to predict over 17,000 unlabeled genes. For each labeled gene, the risk score was computed by prediction model that left the gene in the hold-out set in each CV.

### Autism Risk Gene Validation Using Differential Gene Expression Evidence

Based on our gene ranking system, we classified genes into risk and non-risk genes using a threshold of risk score of 0.22 (resulting in 1,109 predicted ASD genes). We chose the risk score threshold of 0.22 because it gave the highest prediction accuracy (F_1_ score = 0.59) on training data. Genes with a risk score higher than the threshold were predicted as risk genes and the remaining genes were predicted as non-risk genes. We validated the classification performance by examining whether our predicted risk genes show DGE evidence for ASD. Specifically, we obtained DGE summary statistics (beta and *p*-values) for ASD from RNA-Seq datasets for four major cortical lobes (frontal, temporal, parietal, and occipital) and their average from Supplementary Table S1 of a previous study (Gandal et al., 2018), as well as the summary statistics for a non-psychiatric disorder inflammatory bowel disease (IBD) and two psychiatric disorders (bipolar disorder and schizophrenia) that we employed as negative controls from the same study (Gandal et al., 2018). The DGE summary statistics for IBD was derived using a linear mixed-effect model from meta-analysis of two published gene-expression microarray studies. The DGE summary statistics for ASD, bipolar disorder and schizophrenia were calculated using *limma* (Ritchie et al., 2015) with empiric Bayes moderated t-statistics from RNA-Seq analyses of post-mortem brain samples. The details for each datasets and DGE analyses were provided in the original study (Gandal et al., 2018). We used simulation-based approach to estimate the enrichment statistics of predicted risk genes in DGE evidence. We first generated a background distribution from 100,000 random gene sets, while matching for gene size found in predicted risk genes. The enrichment fold was estimated by the ratio of the observed number of risk genes with DGE evidence (*p* < 0.05) to the average number of that from random gene sets. The *p*-value for enrichment was then the proportion of random gene sets with the same or a greater number of genes with DGE evidence, as compared to the number found for predicted risk genes. To investigate whether the enrichment of DGE evidence was specific to ASD, we also performed the same enrichment analysis for IBD, bipolar disorder, and schizophrenia.

### Autism Risk Gene Validation in Independent Sequencing Studies

We further evaluated our gene ranking system utilizing genes targeted by *de novo* LoF mutations from two studies, including one that performed whole exome sequencing of 2,517 families in the Simons Simplex Collection (SSC) cohort (Iossifov et al., 2014) and another that performed whole genome sequencing of the MSSNG cohort (Ryan et al., 2017). To get independent lists of genes for validation, we excluded candidate genes from the two validation cohorts that overlapped the true positive genes in the training sample. For the SSC cohort, after excluding genes not included in BrainSpan, we compiled a list of 346 singleton LoF *de novo* mutations in probands, and 170 LoF *de novo* mutations in the unaffected siblings as negative controls. From the study of the MSSNG cohort, we created a list of 212 *de novo* LoF mutations in probands, 58 statistically significant *de novo* LoF or missense mutations, and 18 statistically significant *de novo* LoF or missense mutations that were not previously reported. For each of the five gene lists, we tested whether a larger proportion of genes were observed in the first decile of our gene ranking system than expected using a binomial test. The expected proportion (0.166) was determined using the percentage of genes with synonymous *de novo* mutations in the unaffected siblings of the SSC cohort.

### Comparison With Other Ranking Systems

We compared our predictions with five autism gene prediction scores, including the ExAC score (pLI) (Lek et al., 2016), Iossifov probability score (Iossifov et al., 2015), Krishnan probability score (Krishnan et al., 2016), Zhang D score (Zhang and Shen, 2017), and Duda score (Duda et al., 2018). The former two (Iossifov et al., 2015; Lek et al., 2016) were based on measures of gene intolerance to disruptive variations, and the later three (Krishnan et al., 2016; Zhang and Shen, 2017; Duda et al., 2018) were based on machine learning methods that utilize brain-specific network features or cell-type specific gene expression signatures from mouse. Different gene scoring systems were compared in terms of ranking 173 curated candidate genes, including 130 genes with suggestive evidence from the SFARI Gene database (Category 3) and 43 recurrent *de novo* LoF genes discovered in recent studies (Wang et al., 2016; Li et al., 2017; Ryan et al., 2017; Stessman et al., 2017). We compared the overall ranking of candidate risk genes for different gene scoring systems, with a higher ranking (smaller number) indicating a greater likelihood of being ASD risk genes. We also compared the enrichment of candidate genes in the first decile of different gene scoring systems.

### Gene Ontology Enrichment Analysis

We performed GO enrichment analysis to examine whether predicted risk genes were clustered into specific biological processes. Fisher’s exact test was used to test the enrichment of risk genes in GO terms compared to non-risk genes. GO terms were chosen from the GO ontology of biological processes in MSigDB (v5.2) (Subramanian et al., 2005). To facilitate interpretation of the results, we included 2,758 GO terms that overlapped at least 20, but not more than 2,000 genes with our tested genes. Bonferroni correction was applied for multiple testing correction. Because GO terms were often highly overlapping in genes, we used hierarchical clustering to group significant gene sets into clusters based on similarity of their gene profiles (Chen et al., 2014). We first defined a gene overlapping matrix by counting the number of overlapping genes for each pair of gene sets. The Pearson correlation coefficient *R* was then calculated for each pair of gene sets based on their overlap profiles. The distance matrix for hierarchical clustering was then 1 - *R*. Hierarchical clustering was performed using the “ward” method implemented in the R function “hclust.” The dendrogram and heatmap were plotted using the R function “heatmap.2.”

## Results

### An Overview of Study

**FIGURE 1:**
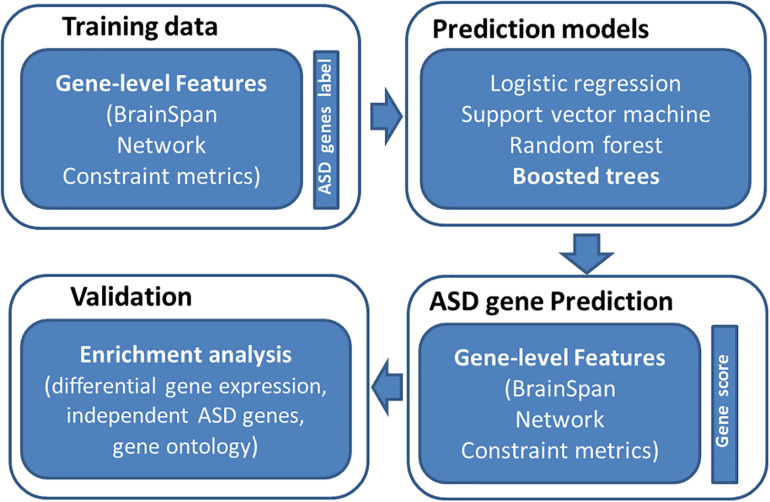
Overview of study design. We first collected training data of labeled genes (121 true positive genes and 963 true negatives) and their associated features (spatiotemporal gene expression values from BrainSpan, network features, and gene-level constraint metrics). We then applied four machine learning algorithms to predict ASD genes, including logistic regression, support vector machine, random forest, and boosted trees. The boosted trees achieved the best prediction performance and was employed to predict ASD risk genes across the genome. We further evaluated predicted risk genes through enrichment analyses.

An overview of study is provided in Figure 1. The basic premise is that ASD risk genes tend to show distinguishing features, including spatial-temporal gene expression patterns in human brain, gene network features, and gene-level constraint metrics. We reason that machine learning models utilizing those features can differentiate ASD genes from normal ones. To evaluate the performance of our prediction model, we examined if predicted ASD genes were enriched for DGE evidence and independent sets of ASD risk genes. We further performed GO enrichment analysis to understand the biology of predicted ASD genes.

### Genome-Wide Prediction of Autism Risk Genes

We visualized gene expression patterns for 1,084 training genes across various regions and developmental stages of human brain (Supplementary Figure S1). There was a trend for known autism risk genes (left gene panel, red rows) to have higher expression levels than non-risk genes (left gene panel, blue rows). We further tested expression level differences between known risk and non-risk genes for each specific brain region and developmental stage (Supplementary Figure S2). The known autism risk genes showed significantly higher expression levels on average than non-risk genes for all tested brain regions and developmental stages (*p* < 0.05). Of note, the difference was stronger for early to middle prenatal stages, ranging from 12 to 21 postconceptional weeks (pcw).

We compared known autism risk and non-risk genes in their sensitivity to mutational changes and other gene variation features. As shown in Supplementary Figure S3, compared to non-risk genes, autism risk genes were more intolerant of missense (mis_z, *p* = 7 × 10^–16^) and LoF mutations (lof_z, *p* = 2 × 10^–23^; pLI, *p* = 2 × 10^–20^), were less likely intolerant of homozygous, but not heterozygous LoF variants (pRec, *p* = 5 × 10^–21^), and had a lower probability of being tolerant of both heterozygous and homozygous LoF variants (pNull, *p* = 3 × 10^–24^). Autism risk genes had longer coding base pairs (*p* = 4 × 10^–29^), a higher probability of mutation across the transcript (mu_syn, *p* = 1 × 10^–16^; mu_mis, *p* = 2 × 10^–18^; mu_lof, *p* = 4 × 10^–19^), and a larger number of rare synonymous or missense variants (n_syn, *p* = 4 × 10^–16^; n_mis, *p* = 1 × 10^–6^), but less number of LoF variants (n_lof, *p* = 3 × 10^–4^).

**FIGURE 2:**
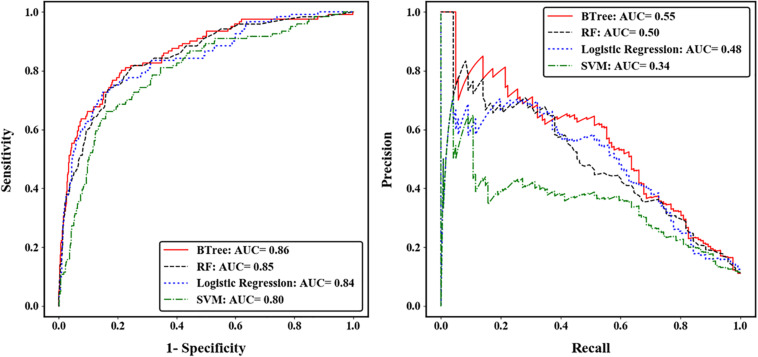
Performance of four machine learning algorithms across five-fold cross validation. The left was measured by the area under receiver operating characteristic curve (ROC), and the right was measured by the area under precision-recall curve (PRC).

We compared the prediction accuracy of four machine learning algorithms across five-fold CV. The gradient boosted trees (BTree) model achieved the best prediction accuracy for autism risk genes with AUC-ROC value of 0.86 and AUC-PRC value of 0.55 (Figure 2). The effects of different features on the boosted trees model were further explored by comparing the prediction accuracy under different feature sets (Supplementary Figure S4). We found that using the spatiotemporal gene expression features alone achieved an AUC-ROC (AUC-PRC) greater than 0.8 (0.4), and that the prediction accuracy was further improved by including either gene network features or gene-level constraint metrics, with the highest accuracy observed when all feature sets were included. We further evaluated the importance of individual features in the optimal BTree model. The feature importance was quantified as the average gain, i.e., improvement in node purity, of the feature when it was used in trees. Supplementary Figure S5 illustrates the top 30 important features, including 28 spatiotemporal expression features and two gene-level constraint metrics (pLI and pNull). It was notable that pLI was the most predictive feature among all features used.

### Autism Risk Gene Validation Using Differential Gene Expression Evidence

**FIGURE 3:**
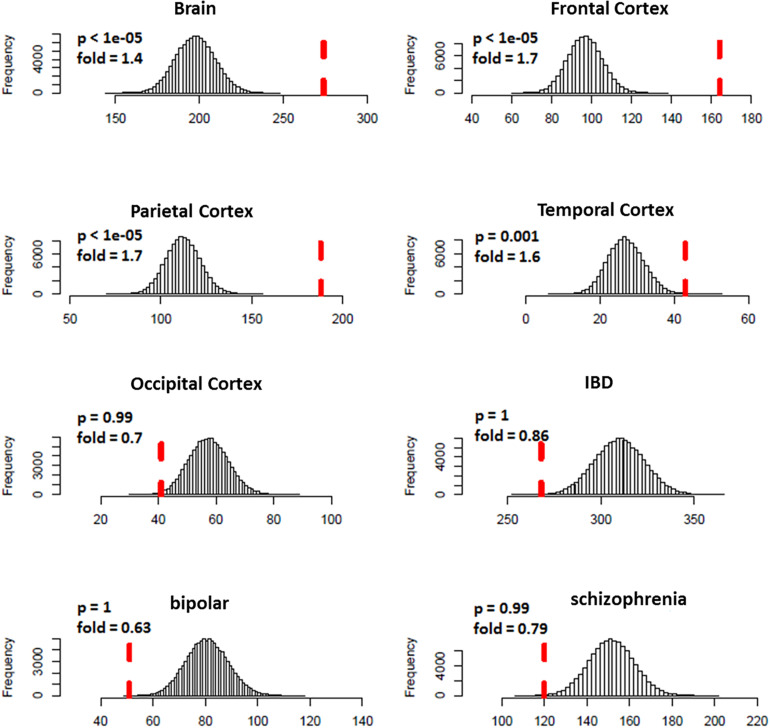
Enrichment analysis of differential expression evidence for predicted ASD risk genes. The histogram shows the distribution for the number of genes with DGE evidence (*p* < 0.05) from random gene sets. The vertical dotted red line indicates the number of genes with DGE evidence from predicted ASD risk genes. Predicted risk genes tended to be down-expressed in brains of ASD, but not for disorders of negative control (IBD, bipolar disorder, and schizophrenia).

We predicted 1,109 risk genes using our gene ranking system under the threshold of risk score > 0.22, which generates the highest prediction accuracy measured by F_1_ score on training data. We then examined whether those predicted risk genes were enriched for DGE evidence for ASD. We found that the predicted risk genes tended to be down-expressed in ASD brains, especially in frontal (fold = 1.7, *p* < 1.0 × 10^–5^) and parietal cortex (fold = 1.7, *p* < 1.0 × 10^–5^) (Figure 3). We did not see any significant enrichment of DGE evidence for IBD, bipolar disorder and schizophrenia, suggesting that the enriched DGE in our predicted genes was specific to ASD.

### Autism Risk Gene Validation in Sequencing Studies

**FIGURE 4:**
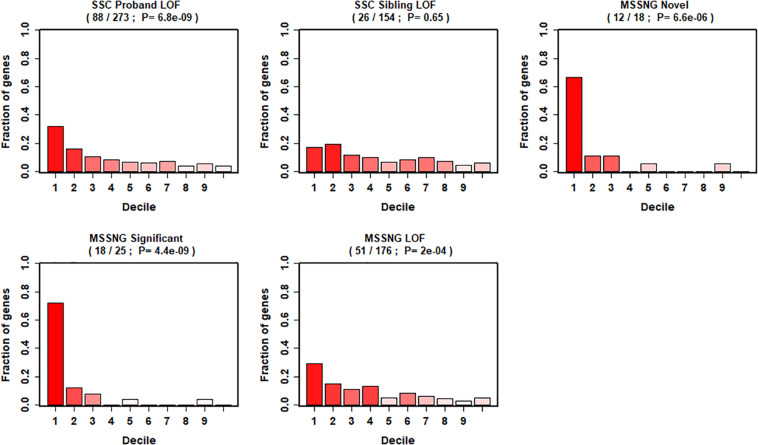
Decile enrichment of *de novo* mutations from two independent cohorts in our gene ranking system.

We further evaluated our gene ranking system using two sequencing studies (Figure 4). For the risk genes identified from the SSC cohort, our top decile genes were significantly enriched with *de novo* LoF mutations in probands. Specifically, genes in the first decile of our ranking system included 32% (88 of 273, *p* = 6.8 × 10^–9^) of *de novo* LOF mutations in probands. In contrast, we did not observe significant enrichment of genes with *de novo* LOF mutations in the unaffected siblings (*p* = 0.65). Similarly, for risk genes identified from the MSSNG cohort, we found significant enrichment for all three gene lists, including the *de novo* LOF mutations in probands (29%, *p* = 2 × 10^–4^), the 25 genes that reached genome-wide significance (72%, *p* = 4.4 × 10^–9^), and the 18 novel genes (67%, *p* = 6.6 × 10^–6^).

### Comparison With Other Ranking Systems

We compared the performance of our ranking system (BTree) with five other gene scoring systems in their ability to rank curated candidate genes. When we examined the rank of an independent set of 173 autism candidate genes, our method outperformed other methods, because our method had the smallest median ranking (indicating the greatest likelihood of the set containing autism risk genes) (Supplementary Figure S6). We further compared the enrichment of 173 candidate genes in the first decile of each gene ranking system (Supplementary Figure S7). We observed the highest proportion of candidate genes in the first decile of our ranking system (52%), which was higher than the Duda score (40%), ExAC score (44%), Iossifov probability score (23%), Krishnan probability score (38%), and Zhang D score (30%). The superior performance of our method might be attributable to the human brain spatiotemporal gene expression features that were not considered in other methods.

### Gene Ontology Enrichment Analysis

We conducted GO enrichment analysis to examine whether predicted 1,109 risk genes (score > 0.22) were clustered into specific biological processes. The full results of this analysis are shown in Supplementary Table S2. There were 179 GO terms that remained significant after Bonferroni correction (*p*_corrected_ < 0.05). Significant GO terms were grouped into five major clusters using hierarchical clustering (Supplementary Figure S8). These clusters included GO terms related to neuronal signaling (orange), neurogenesis (blue and black), chromatin remodeling (green), and transcriptional regulation (red). Table 1 shows details for the top 10 enriched GO terms in enrichment fold that were particularly interesting, as they included GO terms involved in ionotropic glutamate receptor signaling, motor neuron axon guidance, and regulation of histone methylation.

**TABLE 1:** Top ten enriched GO terms in predicted ASD risk genes.

**GO terms**	**OR**	**95%_CI_L**	**95%_CI_U**	***p***	**p_adj_**
GO_CENTRAL_NERVOUS_SYSTEM_PROJECTION_NEURON_AXONOGENESIS	27.0	10.0	80.1	1.7E-11	4.7E-08
GO_IONOTROPIC_GLUTAMATE_RECEPTOR_SIGNALING_PATHWAY	17.5	6.9	45.3	1.9E-09	5.1E-06
GO_CENTRAL_NERVOUS_SYSTEM_NEURON_AXONOGENESIS	17.2	7.1	42.5	4.3E-10	1.2E-06
GO_DENDRITE_MORPHOGENESIS	13.0	6.2	27.0	6.5E-11	1.8E-07
GO_MOTOR_NEURON_AXON_GUIDANCE	11.2	4.4	27.6	6.7E-07	0.0018
GO_POSITIVE_REGULATION_OF_HISTONE_METHYLATION	11.1	5.0	24.4	1.4E-08	4.0E-05
GO_GLUTAMATE_RECEPTOR_SIGNALING_PATHWAY	11.1	5.4	22.4	3.1E-10	8.7E-07
GO_EXCITATORY_POSTSYNAPTIC_POTENTIAL	10.4	4.1	25.2	1.1E-06	0.003
GO_REGULATION_OF_HISTONE_H3_K4_METHYLATION	10.4	4.1	25.2	1.1E-06	0.003
GO_MODULATION_OF_EXCITATORY_POSTSYNAPTIC_POTENTIAL	9.3	3.6	23.2	7.1E-06	0.019

*OR: odds ratio; 95%_CI_L: OR 95% confidence interval lower bound; 95%_CI_U: OR 95% confidence interval upper bound.*

## Discussion

A number of methods have been developed for inferring ASD risk genes. Although they employ differing computational methodologies, most methods were built upon the concept of guilt-by-association, using the assumption that risk genes are functionally related. Theoretically, ASD risk genes should exert their effects at specific developmental stages in specific brain tissues or cell types that are critical to disease development. However, most existing methods have not considered the spatial and temporal patterns of gene relationships during brain development. In addition, gene-level constraint metrics, such as loss of function intolerance, have been used to prioritize ASD candidate genes, but no studies have quantitatively examined their potential for predicting ASD genes. Employing a supervised machine learning algorithm, we have shown that a combination of human brain spatiotemporal gene expression patterns and the gene-level constraint metric features predict ASD risk genes. We further demonstrated the validity of our method through validations using DGE evidence and independent sets of risk genes. We have further shown the superior performance of our ranking system over several other state-of-the-art ranking systems in ranking curated candidate genes.

We explored the potential role of the top ranked genes in ASD risk. The gene *NBEA*, which encodes neurobeachin that is a brain-specific kinase-anchoring protein implicated synaptic structure and function, was assigned the highest probability for conferring ASD risk (score = 0.97). Indeed, mutations in *NBEA* have been identified in ASD (Castermans et al., 2003; Wise et al., 2015) and neurodevelopmental disorders (Mulhern et al., 2018). Another notable gene in our top list was *HERC1* (ranked third, score = 0.94), which encodes a protein that is a probable E3 ubiquitin-protein ligase. Multiple lines of evidence indicate a role for *HERC1* in ASD: (1) it was reported that *HERC1* mutations caused intellectual disability and facial dysmorphism in two Colombian siblings (Ortega-Recalde et al., 2015); (2) A nonsense variant in *HERC1* was associated with intellectual disability, megalencephaly, thick corpus callosum and cerebellar atrophy (Nguyen et al., 2016); (3) importantly, mutations in *HERC1* were reported to be associated with ASD in an exome sequencing study (Hashimoto et al., 2016). Our ranking system also successfully predicted another two ASD candidate genes *TCF20* (ranked 26th, score = 0.87) and *FBXO11* (ranked 19th, score = 0.88). Intriguingly, *TCF20* was one of the highest ranking candidate autism risk genes (category 2) according to the most recent version of the SFARI Gene resource. Mutations in *TCF20* were also implicated in Phelan-McDermid syndrome (Upadia et al., 2018), developmental disorders (Deciphering Developmental Disorders Study, 2017), and schizophrenia (Smeland et al., 2017). *FBXO11* was prioritized as a strong ASD candidate gene (Ji et al., 2016), and was recently reported to be associated with a variable neurodevelopmental disorder (Gregor et al., 2018).

Our ranking system also highlighted some potential novel candidate genes that may deserve further investigation. Four genes, *ZYG11B*, *HECTD1*, *CAND1*, and *MYCBP2*, ranked second, fourth, seventh and tenth, are all involved in protein ubiquitination, which has been implicated in neuronal function and brain disorders, including ASD (Mabb and Ehlers, 2010). To our knowledge, direct genetic links between these genes with ASD have not been found. Of note, *CAND1* encodes an essential regulator of Cullin-RING ubiquitin ligases that play a critical role in ubiquitination and protein degradation (Zheng et al., 2002); *MYCBP2* encodes an E3 ubiquitin-protein ligase that plays a role in axon guidance and synapse formation in the developing nervous system. We have provided the whole list of ranked genes with their probability scores in Supplementary Table S3. Researchers can further explore the top-ranked genes or genes of their own interest.

Our study not only provides hundreds of new ASD candidate genes with evidence for involvement in ASD, but also shows that the predicted risk genes are biologically meaningful and are clustered around biological processes relevant to ASD. GO enrichment analysis demonstrated that the predicted risk genes were enriched in GO terms related to neuronal signaling, neurogenesis, chromatin remodeling, and histone modification, all of which are important biological processes implicated in ASD. In addition, among our top 10 ranked genes, we found that five were related to the protein ubiquitination pathway (*HERC1*, *CAND1*, *ZYG11B*, *HECTD1*, and *MYCBP2*), which is consistent with the significant enrichment of protein ubiquitination process in our GO enrichment analysis (GO_PROTEIN_UBIQUITINATION, OR = 2.3, *p*_corrected_ = 1.9 × 10^–6^), supporting the merging role of ubiquitin signaling in ASD (Mabb and Ehlers, 2010; Cheon et al., 2018). Our analyses also highlighted other biological mechanisms that may underlie ASD. For example, there is evidence for roles of RNA alternative splicing (Parikshak et al., 2016) in ASD, which was represented in our top enriched GO terms (GO_RNA_SPLICING, OR = 3.5, *p*_corrected_ = 8.0 × 10^–12^).

Our study also sheds light on when and where ASD genes may exert their effects during brain development. Of the 28 gene expression features from the top 30 important features in the BTree model, 15 referred to brain regions in the early to mid-prenatal stage (≤24 pcw), reinforcing the important role of early prenatal development in ASD. The involved brain regions include the posteroventral (inferior) parietal cortex (IPC), primary motor cortex (area M1, area 4) (M1C), posterior (caudal) superior temporal cortex (area 22c) (STC), inferolateral temporal cortex (area TEv, area 20) (ITC), medial prefrontal cortex (MFC), cerebellum (CB), dorsolateral prefrontal cortex (DFC), and ventrolateral prefrontal cortex (VFC).

This work should be viewed in light of several limitations. First, our method was trained on genes implicated in ASD by *de novo* mutations. It was not clear how our gene ranking system was relevant to genes affected by other type of variants. Second, our gene ranking system was validated using enrichment analyses of DGE evidence in ASD brain and independent lists of candidate genes. However, a more solid validation should be a replication study for top ranked genes in independent samples through sequencing, but it is beyond the scope of current study. Third, given the strong evidence of clinical and genetic overlap between ASD and other types of neurodevelopmental disorders (Mullin et al., 2013; Srivastava and Schwartz, 2014), further work is needed to investigate whether our gene ranking system is specific to ASD.

In summary, our study has demonstrated that human brain spatiotemporal gene expression patterns and gene-level constraint metrics predict ASD risk genes. Our gene ranking system provides a useful resource for prioritizing ASD candidate genes.

## Data Availability Statement

The datasets generated from this study can be found in online Supplementary Material.

## Author Contributions

SH and AR designed the study and wrote the manuscript. YL performed the data analyses and wrote the manuscript. SA performed the data analyses. AR and JP supported the manuscript preparation and project planning. All authors contributed to the article and approved the submitted version.

## Conflict of Interest

The authors declare that the research was conducted in the absence of any commercial or financial relationships that could be construed as a potential conflict of interest.

## Footnotes

1 https://www.sfari.org/resource/sfari-gene/

2 http://www.brainspan.org/

3 ftp://ftp.broadinstitute.org/pub/ExAC_release/release0.3.1/functional_gene_constraint/fordist_cleaned_exac_r03_march16_z_pli_rec_null_data.txt

## Supplementary Material

FIGURE S1 Heatmap view of spatiotemporal gene expression in human brain. Each cell in the heat map corresponds to the expression level of a gene (row) in a specific brain region and development stage (column). The ASD risk and non-risk genes are denoted by red and blue rows, respectively. Brain regions are represented by the 31 colors in Color Key of Brain Regions. The ASD risk genes tend to be expressed in a higher level compared to non-risk genes across developmental stages and brain regions. The intensity of the color in each cell represents the log2-transformed expression level. Full names of each brain region: CBC, cerebellar cortex; CB, cerebellum; VFC, ventrolateral prefrontal cortex; M1C, primary motor cortex (area M1, area 4); M1C-S1C, primary motor-sensory cortex (samples); IPC, posteroventral (inferior) parietal cortex; PCx, parietal neocortex; HIP, hippocampus (hippocampal formation); DTH, dorsal thalamus; TCx, temporal neocortex; S1C, primary somatosensory cortex (area S1, areas 3,1,2); MD, mediodorsal nucleus of thalamus; A1C, primary auditory cortex (core); AMY, amygdaloid complex; STR, striatum; URL, upper (rostral) rhombic lip; OFC, orbital frontal cortex; Ocx, occipital neocortex; MGE, medial ganglionic eminence; CGE, caudal ganglionic eminence; LGE, lateral ganglionic eminence; STC, posterior (caudal) superior temporal cortex (area 22c); MFC, anterior (rostral) cingulate (medial prefrontal) cortex; V1C, primary visual cortex (striate cortex, area V1/17); ITC, inferolateral temporal cortex (area TEv, area 20); DFC, dorsolateral prefrontal cortex.

FIGURE S2 Gene expression difference between ASD risk and non-risk genes in the spatiotemporal development of human brain. Each cell in the heat map represents the expression level difference (*t*-test) in a specific brain region (column) and development stage (row). The intensity of color represents the log-transformed *p*-value from a *t*-test. The brain regions and stages without gene expression data are marked as black.

FIGURE S3 Boxplot of gene-level constraint metrics and other gene variation features for true positive (TP) and true negative (TN) genes.

FIGURE S4 Boxplot of AUCs under different feature sets for BTree model. The left was measured by the area under receiver operating curve (ROC), and the right was measured by the area under precision-recall curve (PRC).

FIGURE S5 Top 30 important features in the BTree model.

FIGURE S6 Comparison of our gene ranking system (BTree) with five other gene ranking systems on overall rankings of 173 independent candidate genes.

FIGURE S7 Decile enrichment of 173 independent candidate genes for each gene ranking system. The number on the top of each panel represents the number of 173 curated candidate genes appeared in the first decile of each ranking system.

FIGURE S8 Hierarchical clustering of significant GO terms.

TABLE S1 Feature sets included in prediction model.

TABLE S2 GO enrichment analysis for predicted ASD risk genes.

TABLE S3 Gene risk score predicted from Boosted tree model.
