# Supplementary figures and images for: A Machine Learning Approach to Predicting Autism Risk Genes: Validation of Known Genes and Discovery of New Candidates
Source: Front Genet. 2020 Sep 10;11:500064. doi: 10.3389/fgene.2020.500064 (PMC7513695; doi:10.3389/fgene.2020.500064)

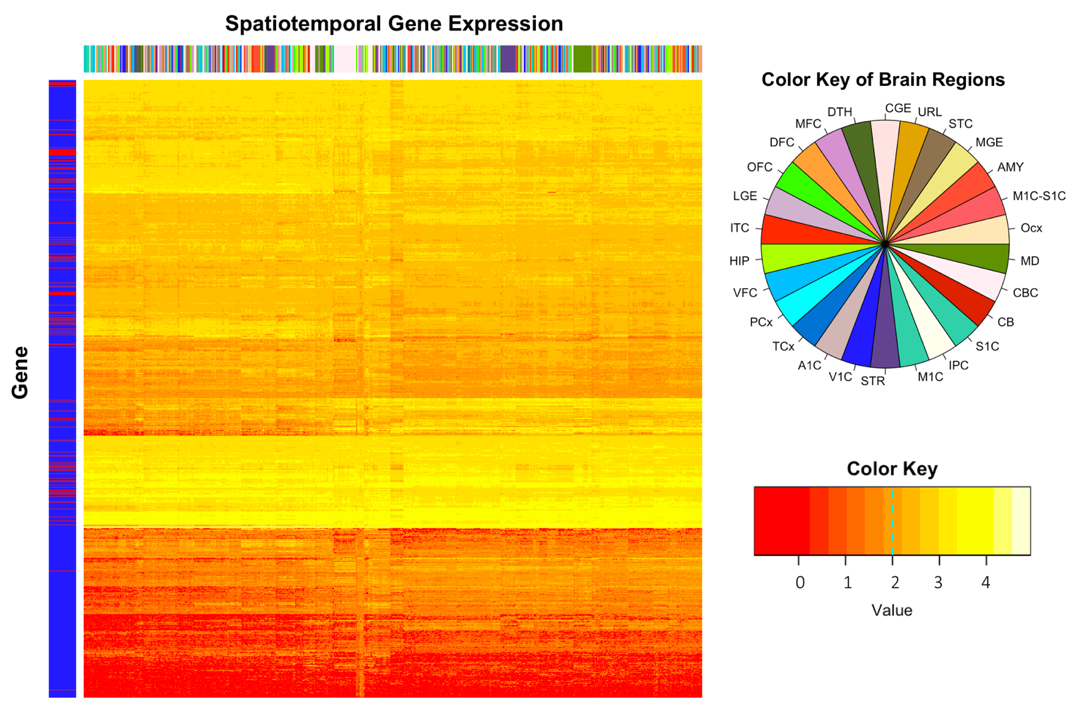

Supplement: FIGURE S1 — Heatmap view of spatiotemporal gene expression in human brain. Each cell in the heat map corresponds to the expression level of a gene (row) in a specific brain region and development stage (column). The ASD risk and non-risk genes are denoted by red and blue rows, respectively. Brain regions are represented by the 31 colors in Color Key of Brain Regions. The ASD risk genes tend to be expressed in a higher level compared to non-risk genes across developmental stages and brain regions. The intensity of the color in each cell represents the log2-transformed expression level. Full names of each brain region: CBC, cerebellar cortex; CB, cerebellum; VFC, ventrolateral prefrontal cortex; M1C, primary motor cortex (area M1, area 4); M1C-S1C, primary motor-sensory cortex (samples); IPC, posteroventral (inferior) parietal cortex; PCx, parietal neocortex; HIP, hippocampus (hippocampal formation); DTH, dorsal thalamus; TCx, temporal neocortex; S1C, primary somatosensory cortex (area S1, areas 3,1,2); MD, mediodorsal nucleus of thalamus; A1C, primary auditory cortex (core); AMY, amygdaloid complex; STR, striatum; URL, upper (rostral) rhombic lip; OFC, orbital frontal cortex; Ocx, occipital neocortex; MGE, medial ganglionic eminence; CGE, caudal ganglionic eminence; LGE, lateral ganglionic eminence; STC, posterior (caudal) superior temporal cortex (area 22c); MFC, anterior (rostral) cingulate (medial prefrontal) cortex; V1C, primary visual cortex (striate cortex, area V1/17); ITC, inferolateral temporal cortex (area TEv, area 20); DFC, dorsolateral prefrontal cortex. [file Image_1.tiff]

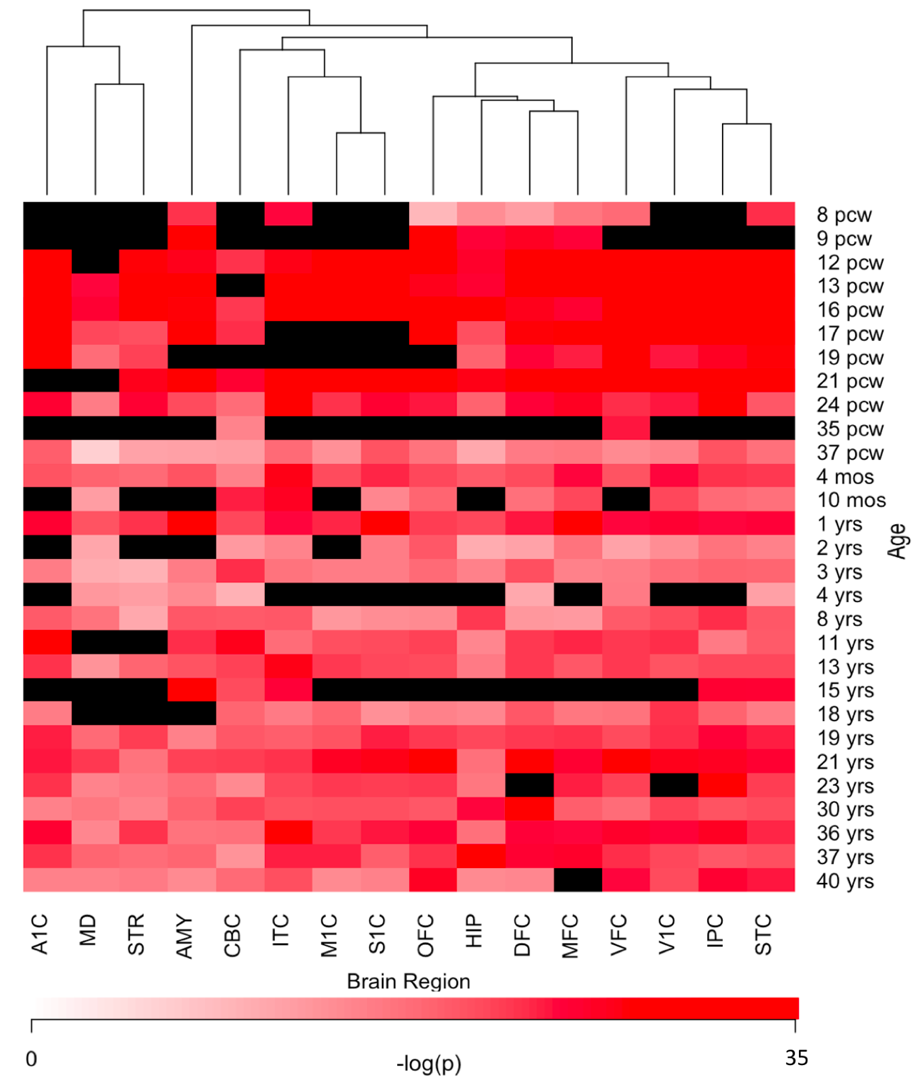

Supplement: FIGURE S2 — Gene expression difference between ASD risk and non-risk genes in the spatiotemporal development of human brain. Each cell in the heat map represents the expression level difference (t-test) in a specific brain region (column) and development stage (row). The intensity of color represents the log-transformed p-value from a t-test. The brain regions and stages without gene expression data are marked as black. [file Image_2.tiff]

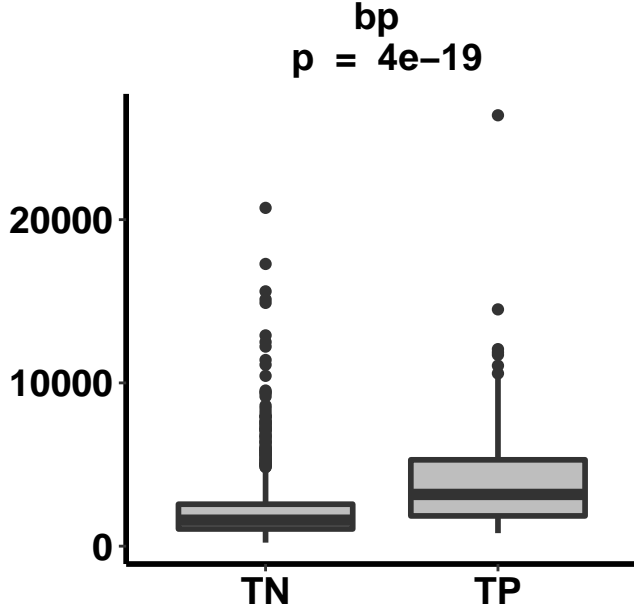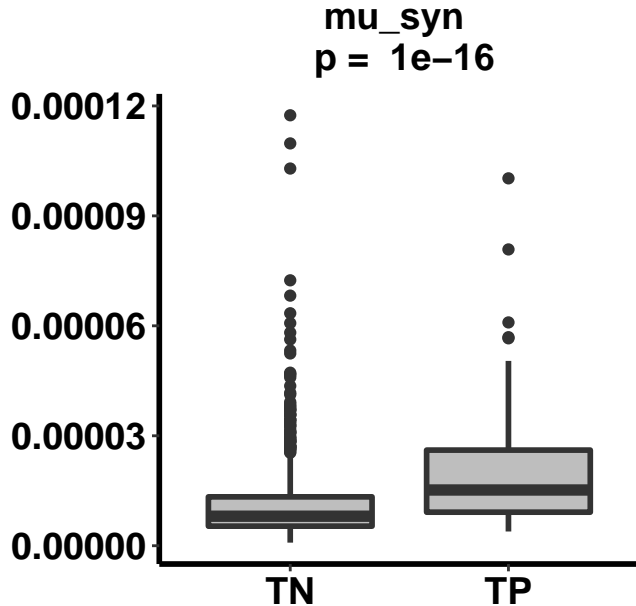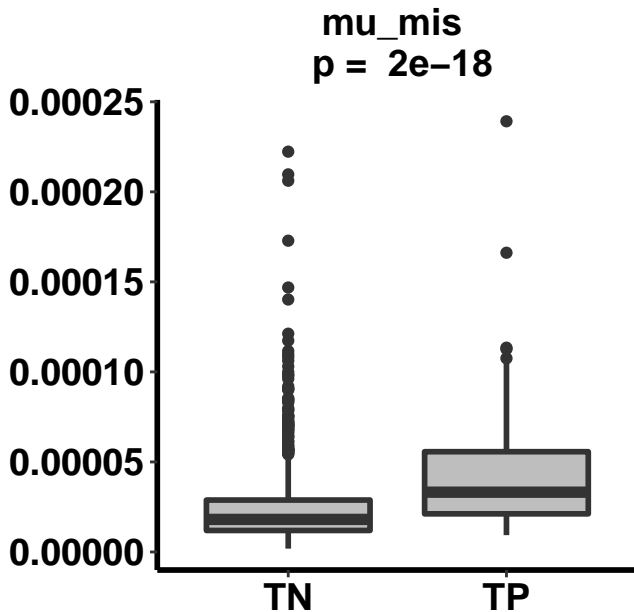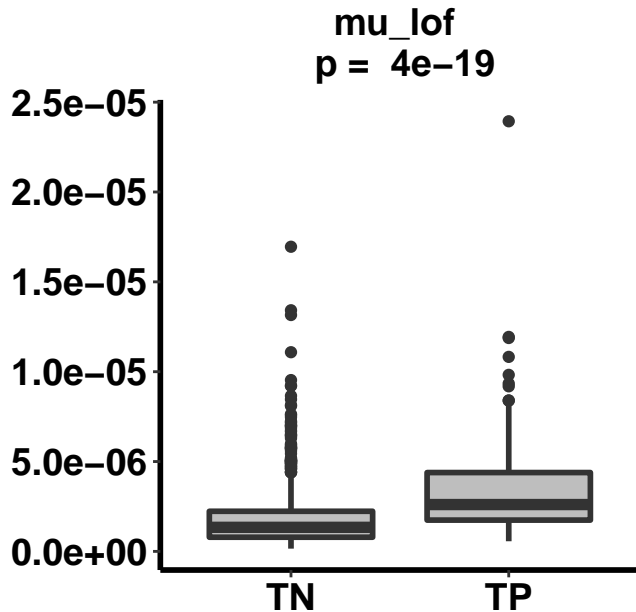

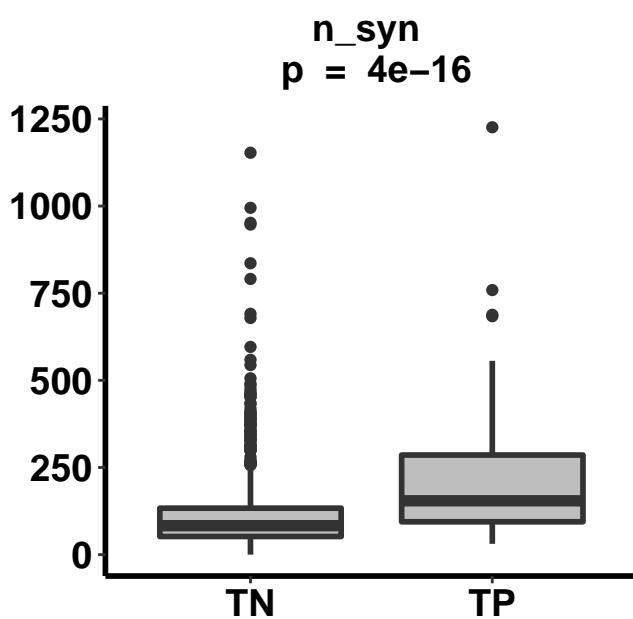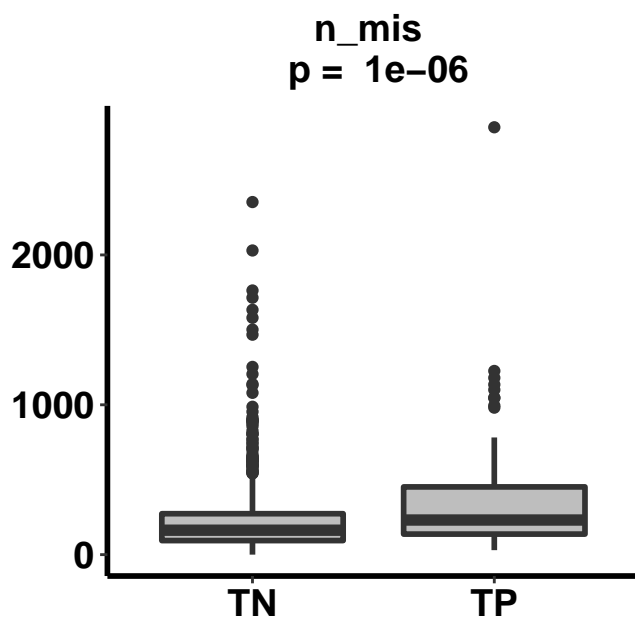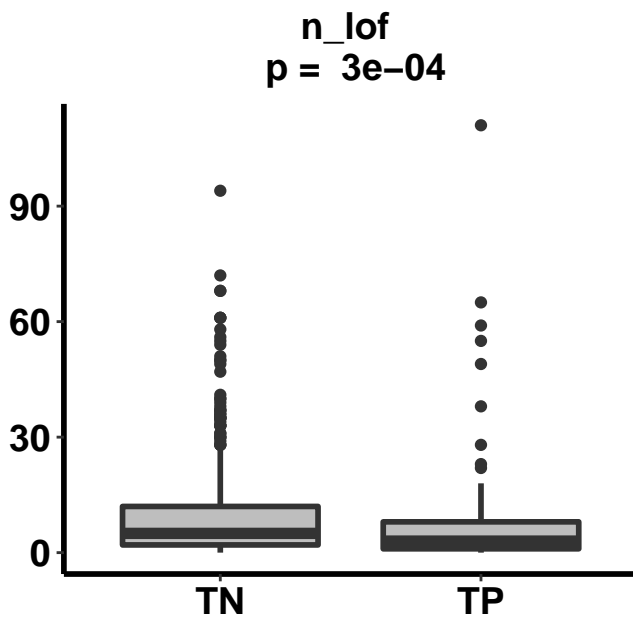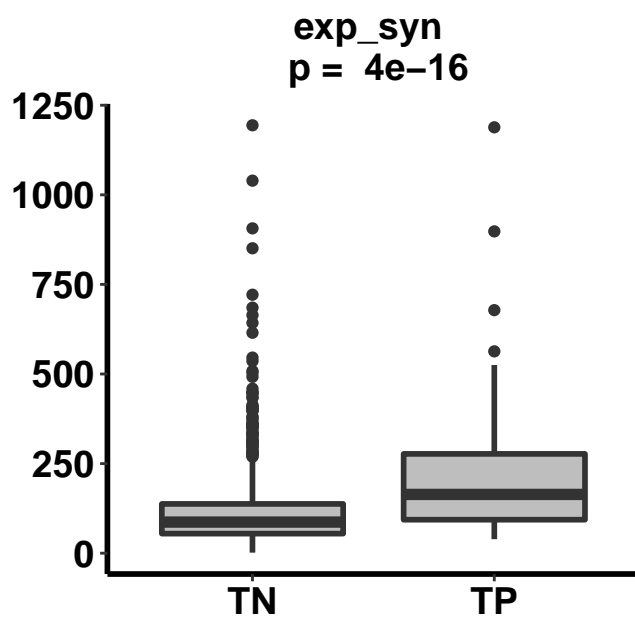

**exp\_mis**  
**p = 2e-17**

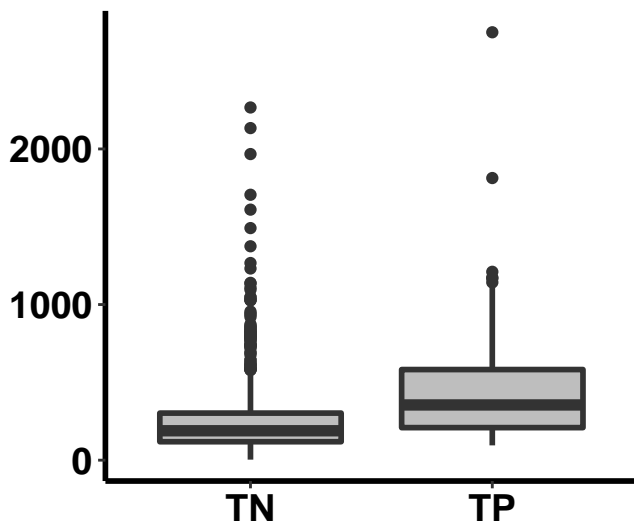

**exp\_lof**  
**p = 6e-17**

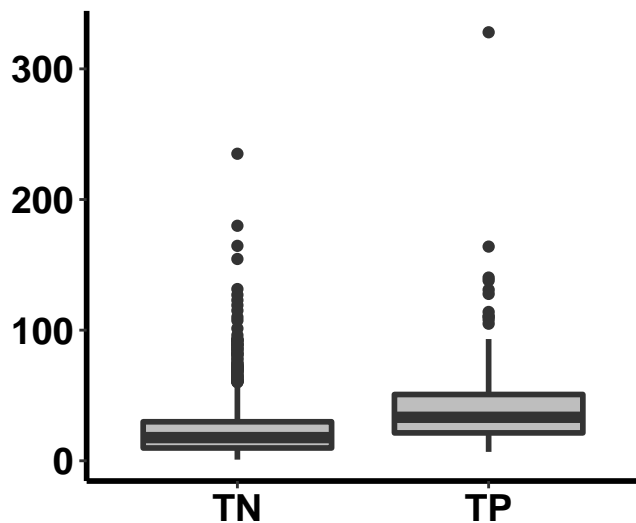

**syn\_z**  
**p = 2e-01**

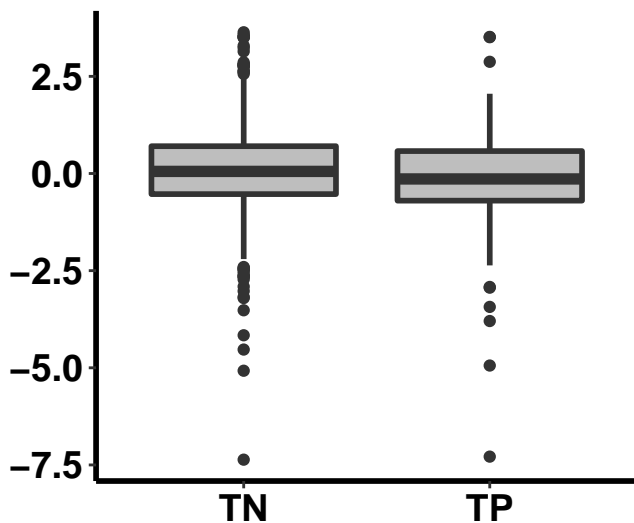

**mis\_z**  
**p = 7e-16**

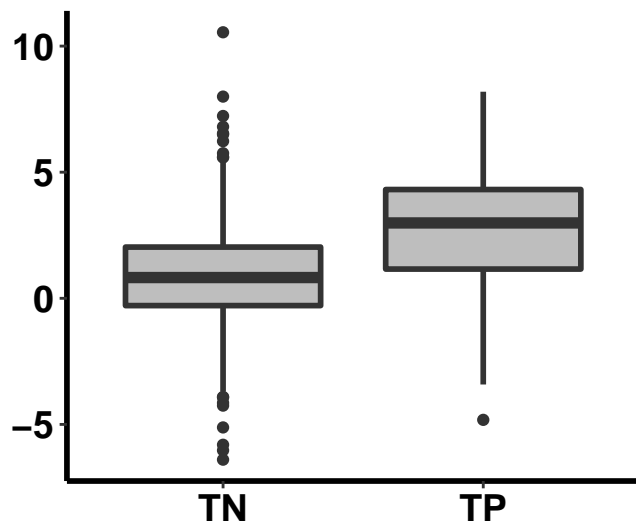

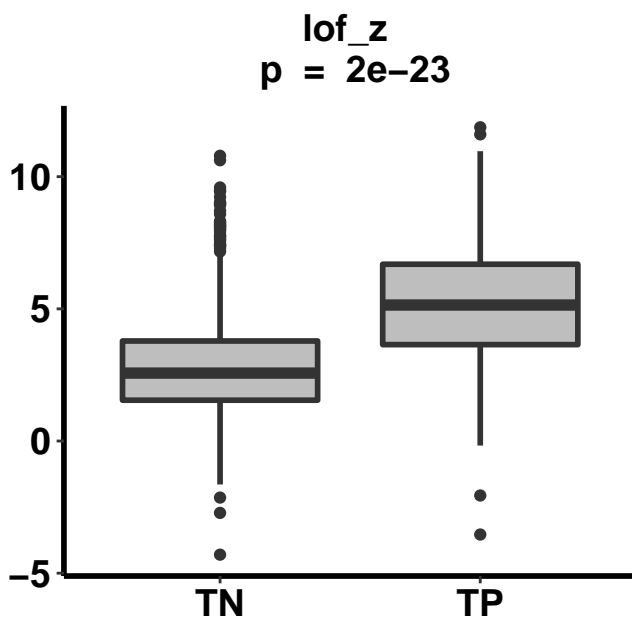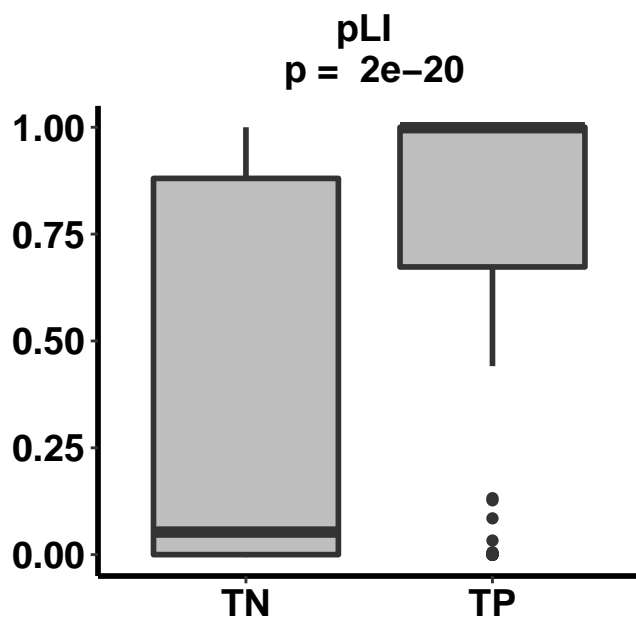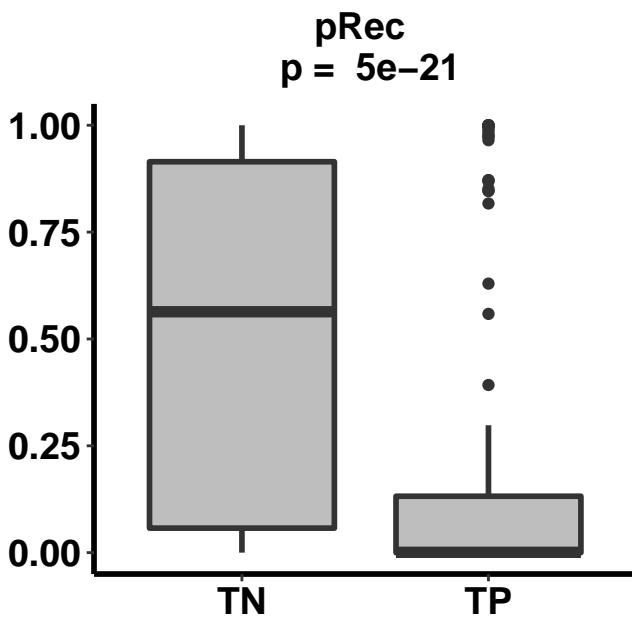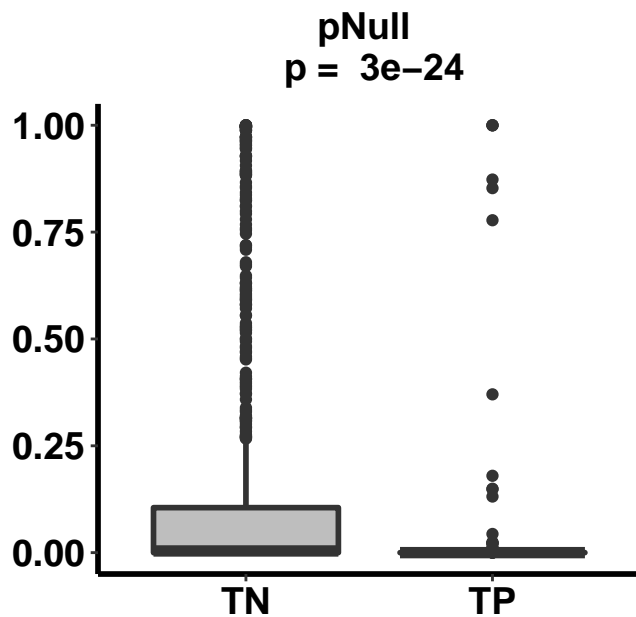

Supplement: FIGURE S3 — Boxplot of gene-level constraint metrics and other gene variation features for true positive (TP) and true negative (TN) genes. [file Image_3.pdf]

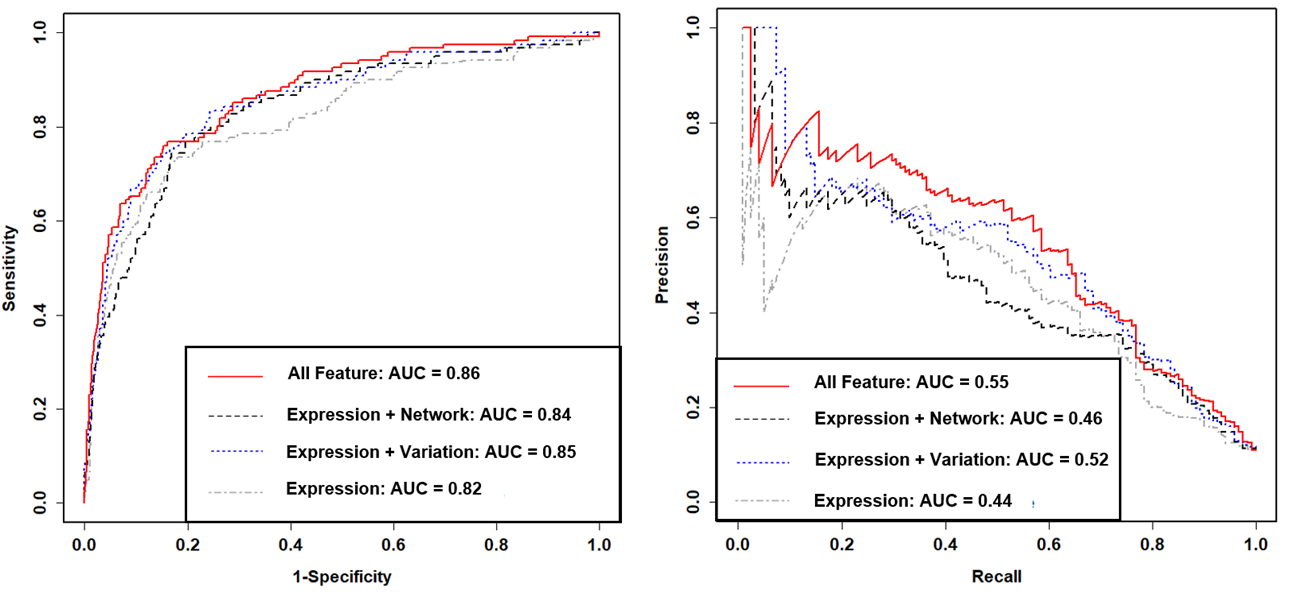

Supplement: FIGURE S4 — Boxplot of AUCs under different feature sets for BTree model. The left was measured by the area under receiver operating curve (ROC), and the right was measured by the area under precision-recall curve (PRC). [file Image_4.tif]

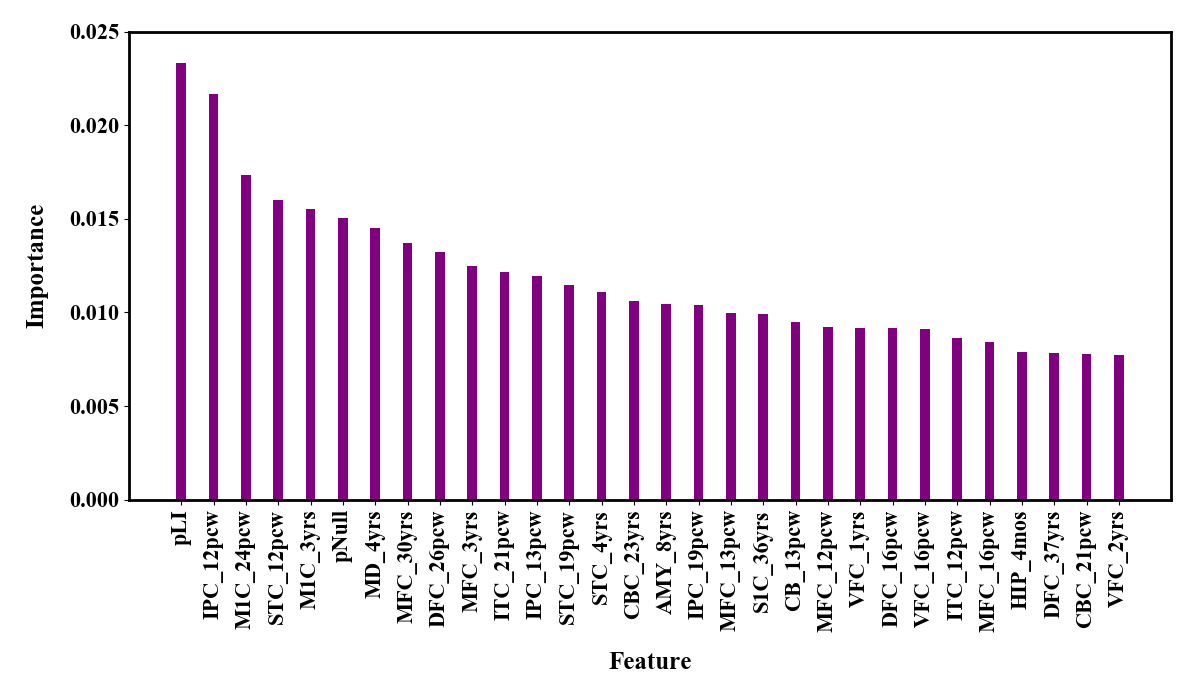

Supplement: FIGURE S5 — Top 30 important features in the BTree model. [file Image_5.tiff]

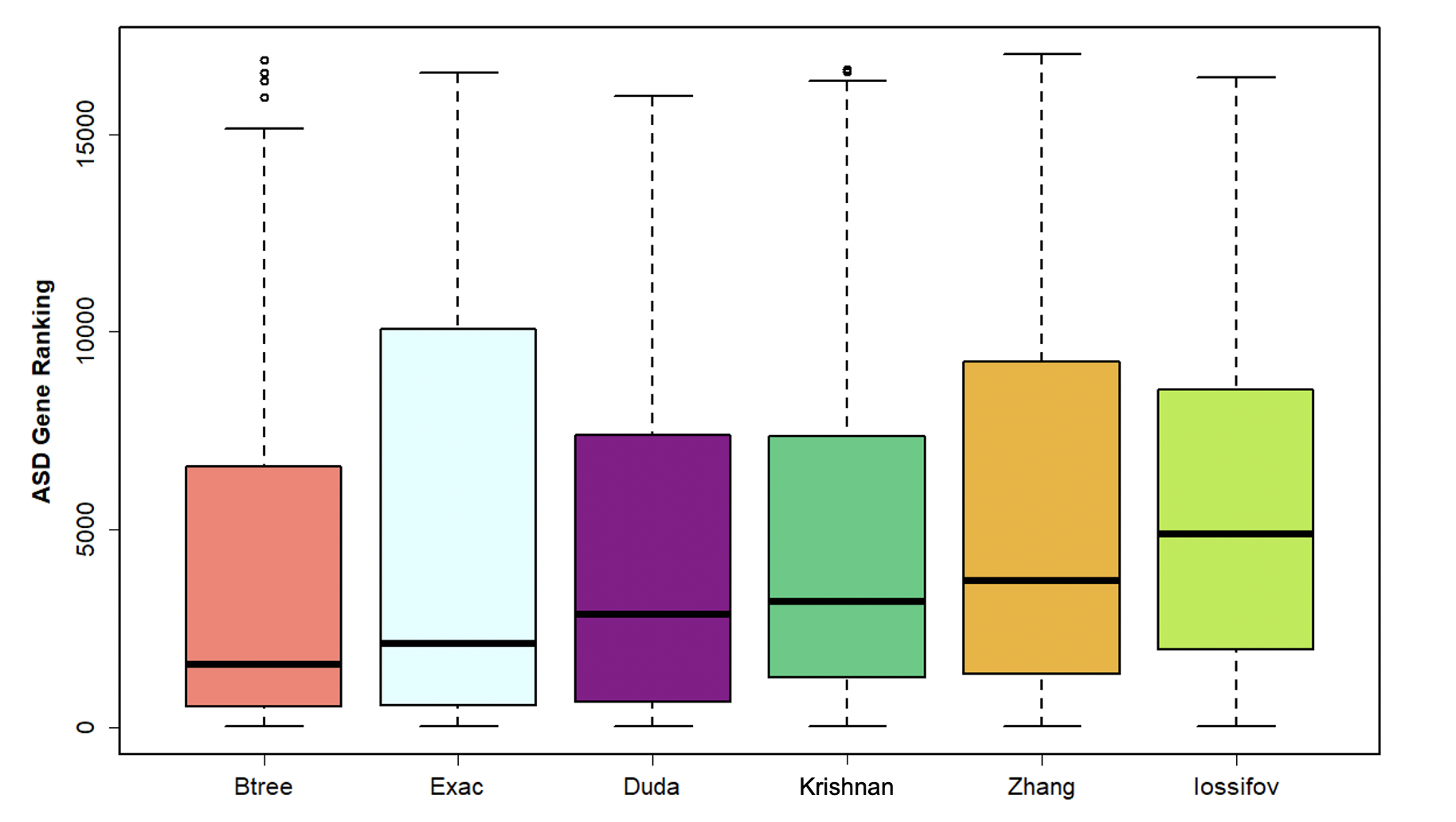

Supplement: FIGURE S6 — Comparison of our gene ranking system (BTree) with five other gene ranking systems on overall rankings of 173 independent candidate genes. [file Image_6.tiff]

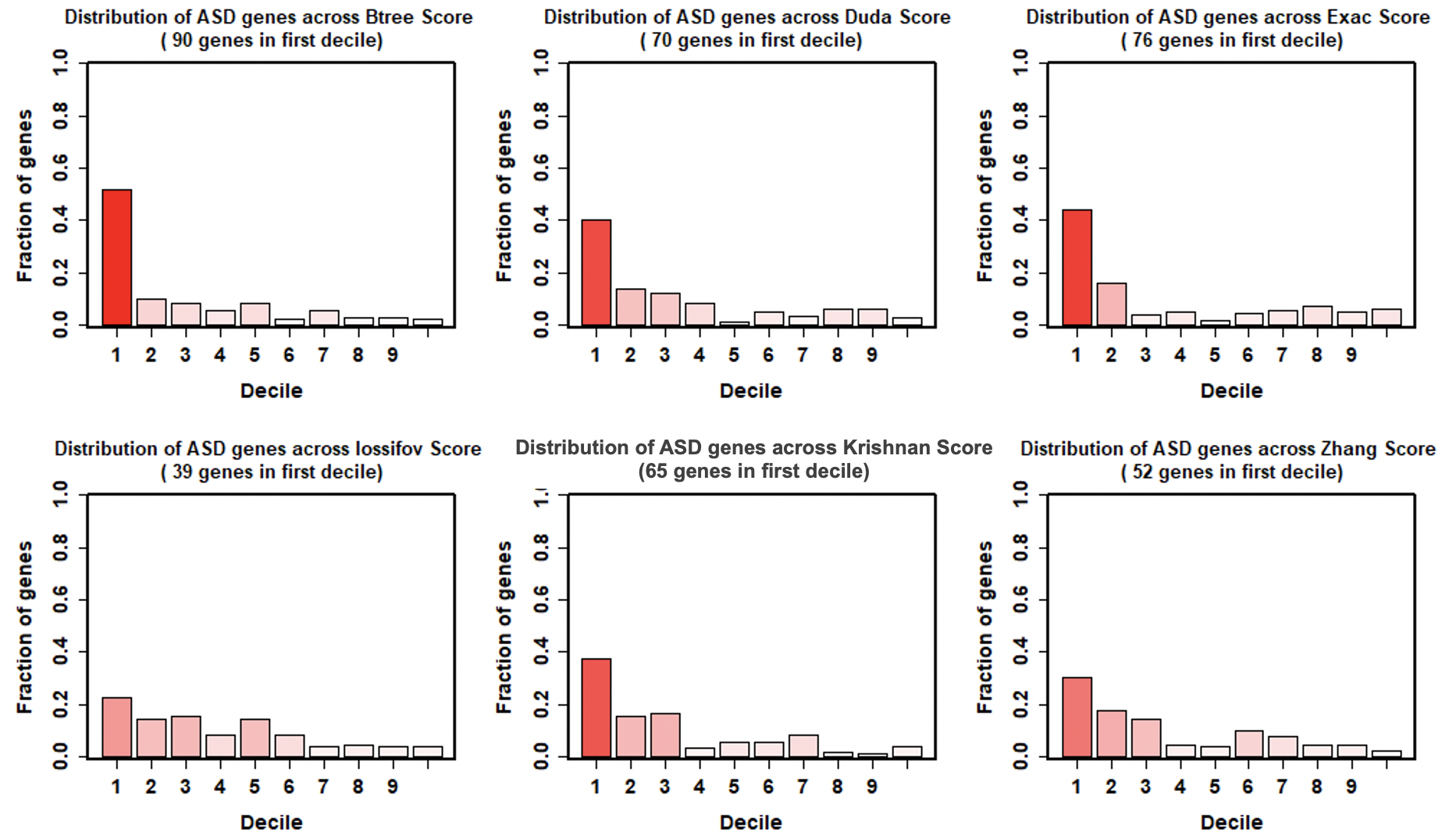

Supplement: FIGURE S7 — Decile enrichment of 173 independent candidate genes for each gene ranking system. The number on the top of each panel represents the number of 173 curated candidate genes appeared in the first decile of each ranking system. [file Image_7.tiff]
