# Supplementary material for: A Machine Learning Approach to Predicting Autism Risk Genes: Validation of Known Genes and Discovery of New Candidates
Source: Front Genet. 2020 Sep 10;11:500064. doi: 10.3389/fgene.2020.500064 (PMC7513695; doi:10.3389/fgene.2020.500064)
Supplement: FIGURE S8 — Hierarchical clustering of significant GO terms. [file Image_8.pdf]

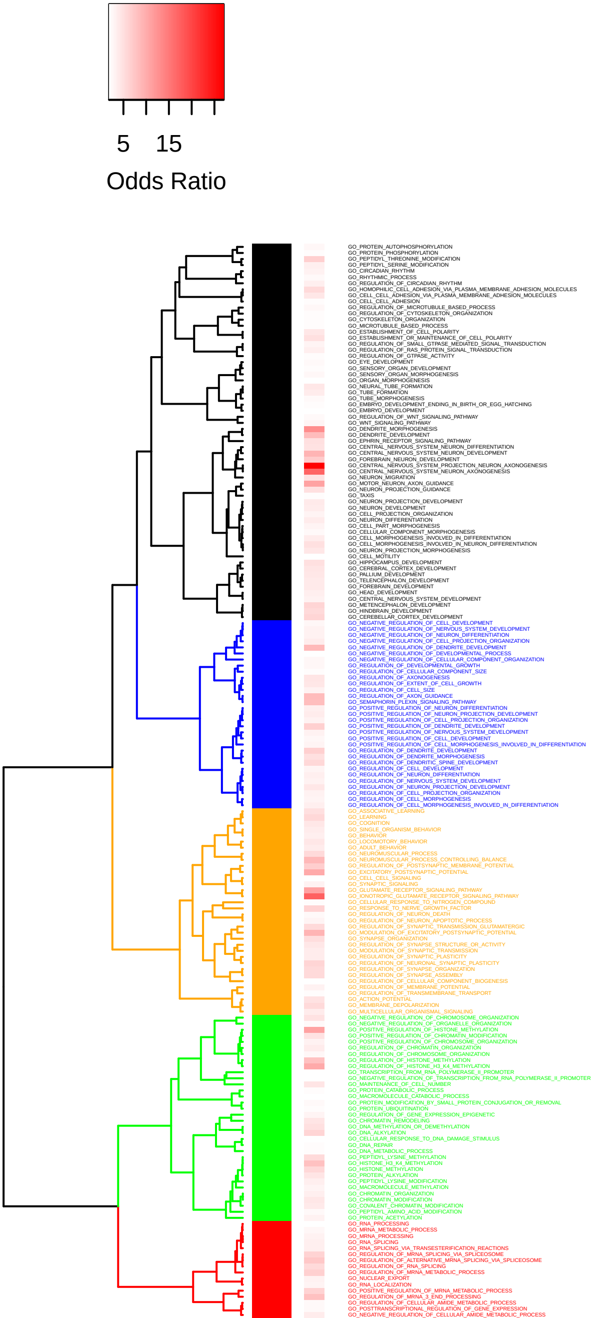

5
15
Odds Ratio
GO_PROTEIN_AUTOPHOSPHORYLATION
GO_PROTEIN_PHOSPHORYLATION
GO_PEPTIDYL_THREONINE_MODIFICATION
GO_PEPTIDYL_SERINE_MODIFICATION
GO_CIRCADIAN_RHYTHM
GO_RHYTHMIC_PROCESS
GO_REGULATION_OF_CIRCADIAN_RHYTHM
GO_HOMOPHILIC_CELL_ADHESION_VIA_PLASMA_MEMBRANE_ADHESION_MOLECULES
GO_CELL_CELL_ADHESION_VIA_PLASMA_MEMBRANE_ADHESION_MOLECULES
GO_CELL_CELL_ADHESION
GO_REGULATION_OF_MICROTUBULE_BASED_PROCESS
GO_REGULATION_OF_CYTOSKELETON_ORGANIZATION
GO_CYTOSKELETON_ORGANIZATION
GO_MICROTUBULE_BASED_PROCESS
GO_ESTABLISHMENT_OF_CELL_POLARITY
GO_ESTABLISHMENT_OR_MAINTENANCE_OF_CELL_POLARITY
GO_REGULATION_OF_SMALL_GTPASE_MEDIATED_SIGNAL_TRANSDUCTION
GO_REGULATION_OF_RAS_PROTEIN_SIGNAL_TRANSDUCTION
GO_REGULATION_OF_GTPASE_ACTIVITY
GO_EYE_DEVELOPMENT
GO_SENSORY_ORGAN_DEVELOPMENT
GO_SENSORY_ORGAN_MORPHOGENESIS
GO_ORGAN_MORPHOGENESIS
GO_NEURAL_TUBE_FORMATION
GO_TUBE_FORMATION
GO_TUBE_MORPHOGENESIS
GO_EMBRYO_DEVELOPMENT_ENDING_IN_BIRTH_OR_EGG_HATCHING
GO_EMBRYO_DEVELOPMENT
GO_REGULATION_OF_WNT_SIGNALING_PATHWAY
GO_WNT_SIGNALING_PATHWAY
GO_DENDRITE_MORPHOGENESIS
GO_DENDRITE_DEVELOPMENT
GO_EPHRIN_RECEPTOR_SIGNALING_PATHWAY
GO_CENTRAL_NERVOUS_SYSTEM_NEURON_DIFFERENTIATION
GO_CENTRAL_NERVOUS_SYSTEM_NEURON_DEVELOPMENT
GO_FOREBRAIN_NEURON_DEVELOPMENT
GO_CENTRAL_NERVOUS_SYSTEM_PROJECTION_NEURON_AXONOGENESIS
GO_CENTRAL_NERVOUS_SYSTEM_NEURON_AXONOGENESIS
GO_NEURON_MIGRATION
GO_MOTOR_NEURON_AXON_GUIDANCE
GO_NEURON_PROJECTION_GUIDANCE
GO_TAXIS
GO_NEURON_PROJECTION_DEVELOPMENT
GO_NEURON_DEVELOPMENT
GO_CELL_PROJECTION_ORGANIZATION
GO_NEURON_DIFFERENTIATION
GO_CELL_PART_MORPHOGENESIS
GO_CELLULAR_COMPONENT_MORPHOGENESIS
GO_CELL_MORPHOGENESIS_INVOLVED_IN_DIFFERENTIATION
GO_CELL_MORPHOGENESIS_INVOLVED_IN_NEURON_DIFFERENTIATION
GO_NEURON_PROJECTION_MORPHOGENESIS
GO_CELL_MOTILITY
GO_HIPPOCAMPUS_DEVELOPMENT
GO_CEREBRAL_CORTEX_DEVELOPMENT
GO_PALLIUM_DEVELOPMENT
GO_TELENCEPHALON_DEVELOPMENT
GO_FOREBRAIN_DEVELOPMENT
GO_HEAD_DEVELOPMENT
GO_CENTRAL_NERVOUS_SYSTEM_DEVELOPMENT
GO_METENCEPHALON_DEVELOPMENT
GO_HINDBRAIN_DEVELOPMENT
GO_CEREBELLAR_CORTEX_DEVELOPMENT
GO_NEGATIVE_REGULATION_OF_CELL_DEVELOPMENT
GO_NEGATIVE_REGULATION_OF_NERVOUS_SYSTEM_DEVELOPMENT
GO_NEGATIVE_REGULATION_OF_NEURON_DIFFERENTIATION
GO_NEGATIVE_REGULATION_OF_CELL_PROJECTION_ORGANIZATION
GO_NEGATIVE_REGULATION_OF_DENDRITE_DEVELOPMENT
GO_NEGATIVE_REGULATION_OF_DEVELOPMENTAL_PROCESS
GO_NEGATIVE_REGULATION_OF_CELLULAR_COMPONENT_ORGANIZATION
GO_REGULATION_OF_DEVELOPMENTAL_GROWTH
GO_REGULATION_OF_CELLULAR_COMPONENT_SIZE
GO_REGULATION_OF_AXONOGENESIS
GO_REGULATION_OF_EXTENT_OF_CELL_GROWTH
GO_REGULATION_OF_CELL_SIZE
GO_REGULATION_OF_AXON_GUIDANCE
GO_SEMAPHORIN_PLEXIN_SIGNALING_PATHWAY
GO_POSITIVE_REGULATION_OF_NEURON_DIFFERENTIATION
GO_POSITIVE_REGULATION_OF_NEURON_PROJECTION_DEVELOPMENT
GO_POSITIVE_REGULATION_OF_CELL_PROJECTION_ORGANIZATION
GO_POSITIVE_REGULATION_OF_DENDRITE_DEVELOPMENT
GO_POSITIVE_REGULATION_OF_NERVOUS_SYSTEM_DEVELOPMENT
GO_POSITIVE_REGULATION_OF_CELL_DEVELOPMENT
GO_POSITIVE_REGULATION_OF_CELL_MORPHOGENESIS_INVOLVED_IN_DIFFERENTIATION
GO_REGULATION_OF_DENDRITE_DEVELOPMENT
GO_REGULATION_OF_DENDRITE_MORPHOGENESIS
GO_REGULATION_OF_DENDRITIC_SPINE_DEVELOPMENT
GO_REGULATION_OF_CELL_DEVELOPMENT
GO_REGULATION_OF_NEURON_DIFFERENTIATION
GO_REGULATION_OF_NERVOUS_SYSTEM_DEVELOPMENT
GO_REGULATION_OF_NEURON_PROJECTION_DEVELOPMENT
GO_REGULATION_OF_CELL_PROJECTION_ORGANIZATION
GO_REGULATION_OF_CELL_MORPHOGENESIS
GO_REGULATION_OF_CELL_MORPHOGENESIS_INVOLVED_IN_DIFFERENTIATION
GO_ASSOCIATIVE_LEARNING
GO_LEARNING
GO_COGNITION
GO_SINGLE_ORGANISM_BEHAVIOR
GO_BEHAVIOR
GO_LOCOMOTORY_BEHAVIOR
GO_ADULT_BEHAVIOR
GO_NEUROMUSCULAR_PROCESS
GO_NEUROMUSCULAR_PROCESS_CONTROLLING_BALANCE
GO_REGULATION_OF_POSTSYNAPTIC_MEMBRANE_POTENTIAL
GO_EXCITATORY_POSTSYNAPTIC_POTENTIAL
GO_CELL_CELL_SIGNALING
GO_SYNAPTIC_SIGNALING
GO_GLUTAMATE_RECEPTOR_SIGNALING_PATHWAY
GO_IONOTROPIC_GLUTAMATE_RECEPTOR_SIGNALING_PATHWAY
GO_CELLULAR_RESPONSE_TO_NITROGEN_COMPOUND
GO_RESPONSE_TO_NERVE_GROWTH_FACTOR
GO_REGULATION_OF_NEURON_DEATH
GO_REGULATION_OF_NEURON_APOPTOTIC_PROCESS
GO_REGULATION_OF_SYNAPTIC_TRANSMISSION_GLUTAMATERGIC
GO_MODULATION_OF_EXCITATORY_POSTSYNAPTIC_POTENTIAL
GO_SYNAPSE_ORGANIZATION
GO_REGULATION_OF_SYNAPSE_STRUCTURE_OR_ACTIVITY
GO_MODULATION_OF_SYNAPTIC_TRANSMISSION
GO_REGULATION_OF_SYNAPTIC_PLASTICITY
GO_REGULATION_OF_NEURONAL_SYNAPTIC_PLASTICITY
GO_REGULATION_OF_SYNAPSE_ORGANIZATION
GO_REGULATION_OF_SYNAPSE_ASSEMBLY
GO_REGULATION_OF_CELLULAR_COMPONENT_BIOGENESIS
GO_REGULATION_OF_MEMBRANE_POTENTIAL
GO_REGULATION_OF_TRANSMEMBRANE_TRANSPORT
GO_ACTION_POTENTIAL
GO_MEMBRANE_DEPOLARIZATION
GO_MULTICELLULAR_ORGANISMAL_SIGNALING
GO_NEGATIVE_REGULATION_OF_CHROMOSOME_ORGANIZATION
GO_NEGATIVE_REGULATION_OF_ORGANELLE_ORGANIZATION
GO_POSITIVE_REGULATION_OF_HISTONE_METHYLATION
GO_POSITIVE_REGULATION_OF_CHROMATIN_MODIFICATION
GO_POSITIVE_REGULATION_OF_CHROMOSOME_ORGANIZATION
GO_REGULATION_OF_CHROMATIN_ORGANIZATION
GO_REGULATION_OF_CHROMOSOME_ORGANIZATION
GO_REGULATION_OF_HISTONE_METHYLATION
GO_REGULATION_OF_HISTONE_H3_K4_METHYLATION
GO_TRANSCRIPTION_FROM_RNA_POLYMERASE_II_PROMOTER
GO_NEGATIVE_REGULATION_OF_TRANSCRIPTION_FROM_RNA_POLYMERASE_II_PROMOTER
GO_MAINTENANCE_OF_CELL_NUMBER
GO_PROTEIN_CATABOLIC_PROCESS
GO_MACROMOLECULE_CATABOLIC_PROCESS
GO_PROTEIN_MODIFICATION_BY_SMALL_PROTEIN_CONJUGATION_OR_REMOVAL
GO_PROTEIN_UBIQUITINATION
GO_REGULATION_OF_GENE_EXPRESSION_EPIGENETIC
GO_CHROMATIN_REMODELING
GO_DNA_METHYLATION_OR_DEMETHYLATION
GO_DNA_ALKYLATION
GO_CELLULAR_RESPONSE_TO_DNA_DAMAGE_STIMULUS
GO_DNA_REPAIR
GO_DNA_METABOLIC_PROCESS
GO_PEPTIDYL_LYSINE_METHYLATION
GO_HISTONE_H3_K4_METHYLATION
GO_HISTONE_METHYLATION
GO_PROTEIN_ALKYLATION
GO_PEPTIDYL_LYSINE_MODIFICATION
GO_MACROMOLECULE_METHYLATION
GO_CHROMATIN_ORGANIZATION
GO_CHROMATIN_MODIFICATION
GO_COVALENT_CHROMATIN_MODIFICATION
GO_PEPTIDYL_AMINO_ACID_MODIFICATION
GO_PROTEIN_ACETYLATION
GO_RNA_PROCESSING
GO_MRNA_METABOLIC_PROCESS
GO_MRNA_PROCESSING
GO_RNA_SPLICING
GO_RNA_SPLICING_VIA_TRANSESTERIFICATION_REACTIONS
GO_REGULATION_OF_MRNA_SPLICING_VIA_SPLICEOSOME
GO_REGULATION_OF_ALTERNATIVE_MRNA_SPLICING_VIA_SPLICEOSOME
GO_REGULATION_OF_RNA_SPLICING
GO_REGULATION_OF_MRNA_METABOLIC_PROCESS
GO_NUCLEAR_EXPORT
GO_RNA_LOCALIZATION
GO_POSITIVE_REGULATION_OF_MRNA_METABOLIC_PROCESS
GO_REGULATION_OF_MRNA_3_END_PROCESSING
GO_REGULATION_OF_CELLULAR_AMIDE_METABOLIC_PROCESS
GO_POSTTRANSCRIPTIONAL_REGULATION_OF_GENE_EXPRESSION
GO_NEGATIVE_REGULATION_OF_CELLULAR_AMIDE_METABOLIC_PROCESS
